# Pathogenic Mitochondrial DNA Mutations Are Common in the General Population

**DOI:** 10.1016/j.ajhg.2008.07.004

**Published:** 2008-08-01

**Authors:** Hannah R. Elliott, David C. Samuels, James A. Eden, Caroline L. Relton, Patrick F. Chinnery

**Affiliations:** 1 Mitochondrial Research Group, Newcastle University, Newcastle upon Tyne, NE2 4HH, UK; 2 Virginia Bioinformatics Institute, Virginia Polytechnic Institute and State University, Blacksburg, VA 24061, USA; 3 Institute of Human Genetics, Newcastle University, Newcastle upon Tyne, NE2 4HH, UK

## Abstract

Mitochondrial DNA (mtDNA) mutations are a major cause of genetic disease, but their prevalence in the general population is not known. We determined the frequency of ten mitochondrial point mutations in 3168 neonatal-cord-blood samples from sequential live births, analyzing matched maternal-blood samples to estimate the de novo mutation rate. mtDNA mutations were detected in 15 offspring (0.54%, 95% CI = 0.30–0.89%). Of these live births, 0.00107% (95% CI = 0.00087–0.0127) harbored a mutation not detected in the mother's blood, providing an estimate of the de novo mutation rate. The most common mutation was m.3243A→G. m.14484T→C was only found on sub-branches of mtDNA haplogroup J. In conclusion, at least one in 200 healthy humans harbors a pathogenic mtDNA mutation that potentially causes disease in the offspring of female carriers. The exclusive detection of m.14484T→C on haplogroup J implicates the background mtDNA haplotype in mutagenesis. These findings emphasize the importance of developing new approaches to prevent transmission.

## Introduction

Disease-based epidemiological studies provide estimates of the minimum population prevalence of mtDNA disease as ∼1 in 5000,^1^, ^2^, ^3^, ^4^, ^5^, ^6^ but the incidence of new mutations and the frequency of asymptomatic carriers have not been fully established. Fundamental differences between mtDNA inheritance and the Mendelian system mean that de novo mutation rates and carrier frequencies cannot be deduced with a standard Mendelian population-genetic approach. Strict maternal inheritance results in negligible intermolecular recombination apparent at the population level, and the presence of thousands of copies of mtDNA within each diploid mammalian cell adds a further complexity.^7^ Most pathogenic mtDNA mutations are heteroplasmic, with varying amounts of mutated mtDNA present within each cell.^8^ Phenotypic expression is ultimately dependent upon the proportion of mutated mtDNA or the amount of wild-type mtDNA within vulnerable tissues. This only leads to a biochemical defect of the respiratory chain when a critical threshold level is exceeded, with the precise amount varying from mutation to mutation.^8^ In keeping with this, a family with mitochondrial disease usually presents clinically for the first time when an individual inherits a high proportion of mutated mtDNA from a mother who harbors a low level of mutated mtDNA and remains asymptomatic. Such dramatic changes in heteroplasmy can occur in a single generation because of a restriction in the intracellular mtDNA content during embryonic development that is responsible for the mtDNA genetic bottleneck.^9^

Previous estimates of the carrier frequency of mtDNA mutations were largely based on the ascertainment of pedigrees through one or more clinically affected individuals, followed by careful family tracing of the maternal lineage.^1^, ^2^, ^3^, ^4^, ^5^, ^6^ This raises the possibility that de novo mtDNA mutations remain undetected in the population because they are well below the threshold required for phenotypic expression or because they lie below the detection threshold of conventional sequencing. Many of these mutations would be lost through random genetic drift, causing disease only when the percentage level of mutation drifts upwards in one or more family members and exceeds the threshold required for clinical presentation.

A further complication is the strong association between specific pathogenic mtDNA mutations and closely related mtDNA polymorphisms (haplogroups), which is not thought to be due to a founder effect.^10^, ^11^ However, it is not known whether the polymorphisms that define the haplogroup predispose it to de novo mutation events, whether they cause the preferential segregation of pathogenic mutations, or whether they affect clinical expression of the associated disorder. The only way of addressing these issues is by screening a large random sample of the population, with matched maternal samples, to determine whether the detected mutations are de novo or inherited.

## Material and Methods

We measured the frequency of the ten pathogenic mtDNA mutations often found in patients with mitochondrial disease (m.1555A→G, m.3243A→G, m.3460G→A, m.7445A→G, m.8344A→G, m.8993T→G, m.11778G→A, m.13513G→A, m.14459G→A, m.14484T→C) in ∼3000 sequential umbilical-cord-blood samples from north Cumbria in England.^12^ Consent for inclusion into the study exceeded 80%. Ethical approval to investigate mtDNA mutations within this cohort was granted by the West Cumbria Local Research Ethics Committee (Project 381). Previous epidemiological and nuclear genetic studies established that this cohort is a random sample of the population with numerous nuclear alleles in Hardy-Weinberg equilibrium.^13^

### Haplogroup Determination

The ten major European mtDNA haplogroups were determined in 344 random samples from the cohort as described in the literature,^14^ with the use of modified primer sequences. This confirmed that the study group was representative of the UK population (Table 1). Mitochondrial haplogroups were also determined in samples that harbored mtDNA point mutations and in their mothers, with the same approach used.

**Table 1:** mtDNA Haplogroup Distribution in 344 Random Umbilical-Cord-Blood Samples from North Cumbria, England

	**mtDNA Haplogroup**										
	**H**	**U**	**T**	**J**	**W**	**V**	**I**	**X**	**M**	**Other**	**Total**
n	137	79	45	34	14	12	7	7	1	8	344
%	39.83	22.97	13.08	9.88	4.07	3.49	2.03	2.03	0.29	2.33	100
95% CI	34.61–45.21	18.62–27.78	9.70–17.11	6.90–13.54	2.24–6.73	1.82–6.01	0.82–4.15	0.82–4.15	< 0.01–1.61	1.01–4.53	

95% CI = Exact 95% confidence interval. The haplogroup U data includes the K subgroup. There were 27 subjects who belonged to the K subgroup of haplogroup U (K = 7.85% of the entire control cohort, 95% CI = 5.24–11.21).

### High-Throughput Genotyping

The population was genotyped by primer extension of multiplex PCR products with the detection of the allele-specific extension products by matrix-associated laser desorption/ionization time of flight (MALDI-TOF; Sequenom MassARRAY, San Diego, CA). Assays were designed with Sequenom Assay Design software v2.0.7.0, resulting in five multiplex assays. The allelotyping assay was followed according to manufacturer's instructions, with modifications. At the primary PCR step, DNA was amplified under the following conditions: initial denaturation of 95°C for 15 minutes, then 30 cycles of denaturation at 95°C for 20 seconds, annealing at 60°C for 30 seconds, and extension at 72°C for 1 minute. Finally, there was a further extension at 72°C for 3 minutes before the samples were cooled and stored at 4°C. A homogeneous MassEXTEND (hME) reaction mix containing appropriate hME EXTEND mix (1× buffer with 0.225 mM d/ddNTPs), 2.7 μM MassEXTEND primer, and 0.576 U ThermoSequenase (GE Healthcare), made up to a final volume of 2 μL with anH_2_O, was added to each SAP-cleaned PCR product. The microplate was then thermocycled as follows: initial denaturation of 94°C for 2 minutes, then 55 cycles of denaturation at 94°C for 5 seconds, annealing at 52°C for 5 seconds, and extension at 72°C for 5 seconds before cooling to 4°C. The sample microplate and a 384 SpectroCHIP were loaded onto the deck of the Samsung Nanodispenser. 15 nL of solution from the sample microplate was transferred onto the chip, which was read by a Bruker Autoflex Mass Spectrometer system. Data was collected with the use of SpectroACQUIRE v3.3.1.3 software and visualised with the use of MassARRAY Typer v3.4 TyperAnalyzer software. The sensitivity of the MALDI-TOF MS assay for each mutation was assessed with mixed cloned mtDNA fragments in duplicate for both uniplex and multiplex reactions (Table S1, available online).

The assay detected ≥ 10% mutated mtDNA in each case, and for some mutations, the detection threshold was considerably lower (Table S1). For nine mutations, positive calls were confirmed by direct sequencing and last-cycle fluorescent PCR-RFLP from an independent aliquot of DNA, which established the percentage mutated mtDNA (see below). The m.1555A→G mutation was confirmed by cloning and sequencing from a separate aliquot of DNA from the same subject.

### Measurement of mtDNA Heteroplasmy

Positive calls were confirmed in an independent aliquot from the original DNA sample with the use of last-cycle fluorescent PCR-RFLP as described previously.^15^ This also established the percentage mutated mtDNA. For the m.1555A→G mutation, heteroplasmy was quantified by cloning and sequencing of 23 independent mtDNA fragments from an independent aliquot from the original DNA sample. Primers and restriction enzymes and digests are shown in Table S2.

### MtDNA Sequencing

The mitochondrial noncoding control region (D-loop) was sequenced by PCR of four overlapping segments, with the use of forward and reverse M13-tagged primer pairs. Primer sequences are shown in Table S3. PCR products were treated with ExoSAP (ExoSAP-IT, GE Healthcare, USA) and sequenced on a fluorescent genetic analyzer (Beckman-Coulter CEQ 8000) with the standard dideoxy chain-termination method (Beckman-Coulter Quickstart). The sequence data were compared to the revised Cambridge reference sequence (rCRS)^16^ with the use of CEQ Sequence Analysis v2.3.13 analysis software (Beckman-Coulter). This allowed identification of D-loop changes and comparison of positive samples.

### Statistical Analysis

Frequencies were compared by calculation of empirical P-values with a Monte Carlo-based simulation approach based on the method of Roff and Bentzen.^17^ Exact 95% confidence intervals were calculated by the Clopper-Pearson method.^18^

## Results

### Population Prevalence

Known duplicate samples from the same individual were 100% concordant. mtDNA mutations were detected in 15 different subjects (Table 2), giving a total mean frequency of 0.54% for the ten mutations (95% CI = 0.30–0.89%) in neonatal-cord-blood samples. The m.3243A→G mutation was the most common. No positive calls were seen for five mutations: m.7445A→G, m.8344A→G, m.8993T→G, m.13513G→A, and m.14459G→A. In each case, the mutation was confirmed by an independent technique from a fresh aliquot of the source DNA, which was kept in a different laboratory. For the majority of mutations, this was performed with last-cycle fluorescent PCR/RFLP, as described in the Material and Methods section. For m.1555A→G, the presence of the mutation was confirmed in each case by cloning and sequencing 23 independent clones.

**Table 2:** The Frequency of Ten Pathogenic mtDNA Mutations in North Cumbria, England

mtDNA Mutation	1555A→G	3243A→G	3460G→A	7445A→G	8344A→G	8993T→G	11778G→A	13513G→A	14459G→A	14484T→C

Positive Calls	2	4	3	0	0	0	3	0	0	3^a^

	4755-1 T (4.4%),	4394-1 H (32.7%),	2728-1 T (18.4%),				3530-1 K (100.0%),			a4607-1 J (100.0%),
MtDNA Haplogroup^b^	5798-1 J (4.4%)	5218-1 H (10.2%),	2916-1 H (42.5%),				5841-1 H (74.8%),			a0493-1 J (100.0%),
(Percentage of Mutated mtDNA)		5130-1 U (1.7%),	0060-1 H (12.9%)				0282-1 K (56.5%)			2966-1 J (89.1%)
		5025-1 H (0.5%)								

No. of Successful Genotypes	2751	2810	2807	2907	2770	2835	2770	2717	2676	2855

Frequency %	0.07	0.14	0.11	0	0	0	0.11	0	0	0.11
(95% CI)	(0.01–0.26)	(0.04–0.36)	(0.02–0.31)	(0–0.13)	(0–0.13)	(0–0.13)	(0.02–0.32)	(0–0.14)	(0–0.14)	(0.02–0.31)

For nine mutations, the percentage of mutated mtDNA was determined by last-cycle fluorescent PCR. For m.1555A→G, heteroplasmy was quantified by cloning and sequencing of independent mtDNA fragments. The detection threshold for the m.1555A→G mutation could not be quantified because of a lack of an efficient fluorescent RFLP assay. However, dilution curves similar to those demonstrated estimated the assay to be efficient in detecting low levels of heteroplasmy. Quantification by cloning and sequencing of mtDNA fragments of the lowest dilution positively identified by MALDI-TOF MS indicated that the threshold of detection for m.1555A→G was 1.4% (1 of 70 independent clones).

a Two of these three subjects were known to be siblings. CI denotes confidence interval.

b MtDNA haplogroup refers to the individual positive cases and their associated mtDNA haplogroup. The number is the case identifier; the letter refers to the European mtDNA haplogroup. K indicates the K subgroup of haplogroup U.

**Figure 1:**
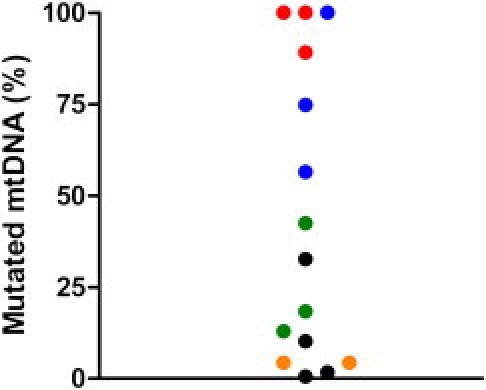
Percentage of Mutated mtDNA in the 15 Mutation-Positive Cases Red: m.14484T→C; blue: m.11778G→A; green: m.3460G→A; black: m.3243A→G; orange: m.1555A→G.

Twelve subjects harbored a heteroplasmic mtDNA mutation. The mean heteroplasmy level for the 15 positive cases was 43% when homoplasmic mutations were included and 29% when homoplasmic mutations were excluded. The percentage level of mutant mtDNA was evenly distributed above and below the mean (Figure 1). The three homoplasmic subjects, including two known siblings, harbored an LHON mutation. D-loop sequencing and mtDNA haplogroup analysis confirmed that other mutation-positive cases were not maternally related to each other or to known pedigrees from the same geographic region, which had a different mtDNA sequence at two or more sites^19^ (Table 3).

**Table 3:** mtDNA D-Loop Sequence for the Mutation-Positive Cases

**Subject ID**	rCRS	**1555A→G**		**3243A→G**								**14484T→C**				**3460G→A**			**11778G→A**				
		4755-1	5798-1	4394-1	**4394-0**^a^	5218-1	**5218-0**^a^	5130-1	**5130-0**^a^	5025-1	**5025-0**^a^	4607-1	**4607-0**^a^	0493-1	02966	02728	02916	00060	3530-1	**3530-0**^a^	5841-1	**5841-0**^a^	00282
**Haplogroup**	H	T	J	H	H	H	H	U	U	H	H	J	J	J	J	T	H	H	K	K	H	H	K
**mtDNA np**																							
22	T	-	-	-	-	C	**C**^a^	-	-	-	-	-	-	-	-	-	-	-	-	-	-	-	-
73	A	G	G	-	-	-	-	G	**G**^a^	-	-	G	**G**^a^	G	G	G	-	-	G	**G**^a^	-	-	G
150	C	-	-	-	-	-	-	T	**T**^a^	-	-	T	**T**^a^	T	-	-	-	-	-	-	-	-	-
152	T	C	-	-	-	-	-	-	-	-	-	C	**C**^a^	C	-	C	-	-	C	**C**^a^	-	-	-
195	T	C	-	-	-	-	-	-	-	-	-	C	**C**^a^	C	-	-	-	-	-	-	-	-	-
200	A	-	-	G	**G**^a^	-	-	-	-	-	-	-	-	-	-	-	-	-	-	-	-	-	-
228	G	-	-	-	-	-	-	-	-	-	-	-	-	-	A	-	-	-	-	-	-	-	-
242	C	-	T	-	-	-	-	-	-	-	-	-	-	-	-	-	-	-	-	-	-	-	-
263	A	G	G	G	**G**^a^	G	**G**^a^	G	**G**^a^	G	**G**^a^	G	**G**^a^	G	G	G	G	G	G	**G**^a^	G	**G**^a^	G
295	C	-	T	-	-	-	-	-	-	-	-	T	**T**^a^	T	T	-	-	-	-	-	-	-	-
309	C	-	-	-	-	-	-	-	-	-	-	-	-	-	-	-	-	-	-	-	-	-	-
309 Ins+1	-	C	-	C	**C**^a^	C	**C**^a^	-	-	-	-	T	**T**^a^	T	-	-	-	C	-	-	C	**C**^a^	-
309 Ins+2	-	-	-	C	-	-	-	-	-	-	-	-	-	-	-	-	-	-	-	-	C	**C**^a^	-
315	C	-	-	-	-	-	-	-	-	-	-	-	-	-	-	-	-	-	-	-	-	-	-
315 Ins+1	-	C	C	C	**C**^a^	C	**C**^a^	C	**C**^a^	C	**C**^a^	C	**C**^a^	C	C	C	C	C	C	**C**^a^	C	**C**^a^	C
319	T	-	-	-	-	-	-	-	-	-	-	C	**C**^a^	C	-	-	-	-	-	-	-	-	-
462	C	-	T	-	-	-	-	-	-	-	-	-	-	-	T	-	-	-	-	-	-	-	-
482	C	-	-	-	-	-	-	-	-	-	-	-	-	-	T	-	-	-	-	-	-	-	-
489	T	-	C	-	-	-	-	-	-	-	-	C	**C**^a^	C	C	-	-	-	-	-	-	-	-
497	C	-	-	-	-	-	-	-	-	-	-	-	-	-	-	-	-	-	T	**T**^a^	-	-	T
513	G	-	-	-	-	-	-	-	-	-	-	A	**A**^a^	A	-	-	-	-	-	-	-	-	-
525	C	-	-	-	-	-	-	-	-	-	-	-	-	-	-	-	-	-	-	-	-	-	-
525 Ins+1	-	-	-	-	-	-	-	-	-	-	-	-	-	-	-	-	-	-	C	**C**^a^	-	-	-
525 Ins+2	-	-	-	-	-	-	-	-	-	-	-	-	-	-	-	-	-	-	A	**A**^a^	-	-	-
525 Ins+3	-	-	-	-	-	-	-	-	-	-	-	-	-	-	-	-	-	-	C	**C**^a^	-	-	-
525 Ins+4	-	-	-	-	-	-	-	-	-	-	-	-	-	-	-	-	-	-	A	**A**^a^	-	-	-
15833	C	-	-	-	-	-	-	-	-	-	-	-	-	-	-	-	-	T	-	-	-	-	-
15928	G	A	-	-	-	-	-	-	-	-	-	-	-	-	-	A	-	-	-	-	-	-	-
16069	C	-	T	-	-	-	-	-	-	-	-	T	**T**^a^	T	T	-	-	-	-	-	-	-	-
16093	T	-	-	-	-	C	**C**^a^	-	-	C	**C**^a^	-	-	-	-	-	-	-	-	-	C	**C**^a^	-
16126	T	C	C	-	-	-	-	-	-	-	-	C	**C**^a^	C	C	C	-	-	-	-	-	-	-
16129	G	-	-	-	-	-	-	-	-	-	-	-	-	-	-	-	-	-	-	-	A	**A**^a^	-
16145	G	-	A	-	-	-	-	-	-	-	-	A	**A**^a^	A	-	-	-	-	-	-	-	-	-
16163	A	G	-	-	-	-	-	-	-	-	-	-	-	-	-	-	-	-	-	-	-	-	-
16172	T	-	C	-	-	-	-	-	-	-	-	-	-	-	-	-	-	C	-	-	-	-	-
16186	C	T	-	-	-	-	-	-	-	-	-	-	-	-	-	-	-	-	-	-	-	-	-
16189	T	C	-	-	-	-	-	-	-	-	-	-	-	-	-	-	-	-	-	-	-	-	-
16192	C	-	T	-	-	-	-	-	-	-	-	-	-	-	-	-	-	-	-	-	-	-	-
16222	C	-	T	-	-	-	-	-	-	-	-	-	-	-	-	-	-	-	-	-	-	-	-
16224	T	-	-	-	-	-	-	-	-	-	-	-	-	-	-	-	-	-	C	**C**^a^	-	-	C
16231	T	-	-	-	-	-	-	-	-	-	-	C	**C**^a^	C	-	-	-	-	-	-	-	-	-
16240	A	-	-	G	**G**^a^	-	-	-	-	-	-	-	-	-	-	-	-	-	-	-	-	-	-
16261	C	-	T	-	-	-	-	-	-	-	-	T	**T**^a^	T	-	-	-	-	-	-	-	-	-
16294	C	T	-	-	-	-	-	-	-	-	-	-	-	-	-	T	-	-	-	-	-	-	-
16296	C	-	-	-	-	-	-	-	-	-	-	-	-	-	-	T	-	-	-	-	-	-	-
16304	T	-	-	-	-	-	-	-	-	-	-	-	-	-	-	C	-	C	-	-	-	-	-
16311	T	-	-	-	-	-	-	-	-	-	-	-	-	-	-	-	-	-	C	**C**^a^	-	-	C
16316	A	-	-	-	-	-	-	-	-	-	-	-	-	-	-	-	-	-	-	-	G	**G**^a^	-
16360	C	-	-	T	**T**^a^	-	-	-	-	-	-	-	-	-	-	-	-	-	-	-	-	-	-
16519	T	C	-	C	**C**	C	**C**^a^	C	**C**^a^	C	**C**^a^	-	-	-	-	C	C	-	C	**C**^a^	C	**C**^a^	C

rCRS indicates nucleotide present in the revised Cambridge reference sequence for mtDNA.^16^ np indicates “nucleotide pair,” numbered according to the rCRS. Dashes indicate rCRS nucleotides.

a Matched maternal samples.

### Mutations Not Present in Maternal Samples

No matched maternal sample was available for seven of the positive cases, but in eight cases it was possible to study a maternal blood sample taken at the time of delivery of the offspring. mtDNA mutations were not detectable in the blood of three mothers, with one harboring m.11778G→A, and two harboring m.3243A→G. This provides an approximation of the de novo mutation for mtDNA defects at 107/100,000 live births (95% CI = 87–127). Two of the mothers, accounting for the three homoplasmic offspring with LHON mutations, were also homoplasmic for the mtDNA mutation.

### MtDNA Haplogroup Association

When the carriers of mtDNA mutations were studied together, the frequency of the major European haplogroups was no different from the background population (Haplogroup H, p = 1.0; J, p = 0.1; U including subgroup K, p = 0.1; T, p = 1). However, all of the m.14484T→C carriers belonged to haplogroup J (p = 0.011, based on a comparison of the two index cases to the background-population data in Table 1). This was not the case for the other two LHON mutations, m.3460G→A and m.11778A→G. Subhaplogroup analysis of the two m.14484T→C pedigrees on the basis of mtDNA D-loop sequencing (Table 3) showed that one family belonged to J2a (siblings 4607-1 and 0493-1 harbored m.16069T, m.16126C, m.150T, m.152C, m. 195C, m.16145A, and m.16231C) and the other to J1c (02966 harbored m.16069T, m.16126C, m.462T, and m.228A but is wild-type at m.152, m16145, m.16222, and m.16261).^11^

## Discussion

Previous studies were based on ascertainment through clinically affected subjects,^1^, ^2^, ^3^, ^4^, ^5^, ^6^ were focused on one specific mutation,^20^ and studied unrelated older subjects^20^ in whom somatic mutation or mutation loss through segregation are potential confounding factors. We studied randomly ascertained neonatal-cord-blood samples in which the percentage level of mutation is likely to be at its highest level.^15^, ^21^, ^22^ By studying ten-point mutations in ∼3000 subjects, we detected a pathogenic mtDNA mutation in > 1/200 live births. It is unlikely that the high frequency of mtDNA mutations detected in the general population in this study was due solely to the use of a sensitive technique, because other mutation-specific studies reported a similar level of sensitivity (∼2–3% for m.3243A→G^2^, ^5^, ^23^). It is also unlikely that our positive results were due to crosscontamination, because we confirmed each result using a different method on an independent aliquot of the source DNA, which was kept in another laboratory; we saw no evidence of heteroplasmy on the haplogroup RFLP assays; and, finally, we saw no heteroplasmic nucleotides on the D-loop sequence trace for each positive case. It is conceivable that, in some subjects, we did not detect an mtDNA mutation because the level of heteroplasmy either fell below the sensitivity of our high-throughput screening assay or was at higher percentage levels in other tissues. Our observations, therefore, provide a minimum figure for the prevalence of ten pathogenic mutations and indicate that mtDNA mutations are amongs the most common pathogenic alleles in the general population.

Although m.7445A→G, m.8344A→G, m.8993T→G, m.13513G→A, and m.14459G→A were not detected, these mutations are also uncommon in mitochondrial-disease patients from the same UK population.^3^, ^6^ By contrast, the most common heteroplasmic mutation was m.3243A→G (33%), reaching frequencies similar to that described in one mutation-specific survey.^20^ m.3243A→G is also the most common heteroplasmic mtDNA mutation in adults with mtDNA disease from the same geographic region (40%).^6^ Therefore, a high mutation rate provides the likely explanation for the prevalence of m.3243A→G in disease cohorts. By contrast, the carrier frequencies of the three major mutations that cause Leber hereditary optic neuropathy (LHON), m.3460G→A, m.11778G→A, and m.14484T→C, were equal. This differs from the distribution for independent families with LHON in the north of England, where the majority of cases harbor the m.11778G→A mutation (60%).^19^ All of the asymptomatic m.14484T→C carriers belonged to mtDNA haplogroup J. This is in keeping with the well-established preferential association between haplogroup J and m.14484T→C LHON pedigrees ascertained through clinically affected individuals.^24^, ^25^ However, the data shown here provide the first direct evidence that this association is not due solely to enhanced clinical expression on J, as previously thought.^10^ Subhaplogroup analysis by mtDNA D-loop sequencing confirmed the independent recurrence of m.14484T→C in asymptomatic carriers within the population, providing independent evidence that haplogroup J predisposes the mitochondrial genome to mutate at np.14484, possibly through near-neighbor effects as previously described to occur during mtDNA evolution.^26^

In the heteroplasmic carriers, heteroplasmy levels were evenly distributed above and below the mean, as expected from population-genetic theory based on neutral alleles.^27^ Of the heteroplasmic offspring with matched maternal samples, the proportion of mutated mtDNA increased with transmission in each case. Although this could be evidence of genetic selection during transmission, the percentage level of m.3243A→G in blood decreases exponentially during life,^15^, ^21^, ^22^ so it is highly likely that some of these mothers originally had higher levels of m.3243A→G, making it difficult to draw firm conclusions. In > 1 in 1000 live births, an mtDNA mutation was present in cord blood from the child but not detectable in the mother's blood, providing an estimation of the de novo mutation rate. It is conceivable that the maternal levels of heteroplasmy were below the detection threshold of the assay, possibly because the level of mutation in blood decreased during the mother's life.^15^, ^21^, ^22^ However, the level of sensitivity for fluorescent PCR-RFLP is in the region of 1.8%,^28^ making this unlikely.

Detecting heteroplasmic mtDNA mutations in >1 in 200 individuals of the background population has implications for studies reporting mtDNA mutations in specific disease groups. Our data show that putative disease associations, such as the reported high frequency of m.3243A→G in diabetes mellitus,^23^, ^29^, ^30^, ^31^ could be a chance finding irrelevant to pathogenesis. We have identified a massive reservoir of pathogenic mtDNA mutations in the general population, placing greater emphasis on developing techniques to prevent the transmission of pathogenic alleles that could segregate to high levels and thus cause mtDNA diseases in subsequent generations.

## Supplemental Data

Document S1. Three Tables
