# Supplementary material for: Pathogenic Mitochondrial DNA Mutations Are Common in the General Population
Source: Am J Hum Genet. 2008 Aug 1;83(2):254–60. doi: 10.1016/j.ajhg.2008.07.004 (PMC2495064; doi:10.1016/j.ajhg.2008.07.004)
Supplement: Document S1. Three Tables [file mmc1.pdf]

Supplemental Data

Pathogenic Mitochondrial DNA Mutations Are Common in the General Population

Hannah R. Elliott, David C. Samuels, James A. Eden, Caroline L. Relton, and Patrick F. Chinnery

Table S1. Nucleotide Base Calls for Specific mtDNA Mutations by MALDI-TOF MS

	**MALDI-TOF MS base call**								
Percentage Heteroplasmy	m.7445A>G	m.3243A>G	m.8344A>G	m.8993T>G	m.13513G>A	m.3460G>A	m.11778G>A	m.14459G>A	m.14484T>C
100.0	G	G	G	G	A	A	A	A	C
88.2					GA				
85.2				TG					
80.2								GA	
77.1									CT
73.2						GA			
72.0			AG						
71.9					GA				
70.5				TG					

65.8	AG								
61.8					GA				
58.8			AG						
53.4					GA				
52.8				TG					
49.7									CT
48.7								GA	
48.7				TG					
47.7									CT
47.3		AG							
47.1								GA	
46.0						GA			
38.2							GA		
35.3	AG								
34.2			AG						
32.1					GA				
29.6				TG					
26.9		AG							
26.6			AG						

25.1				TG					
24.8		AG							
24.8						GA			
24.3								GA	
22.4									CT
22.3						GA			
18.0				TG					
16.9								GA	
15.7	AG								
14.7					GA				
14.2							GA		
13.9						GA			
13.7									CT
13.1						GA			
12.8							GA		
12.0									CT
11.6								GA	
9.8						GA			
9.8							GA		

9.7		AG							
9.7			AG						
9.2								GA	
9.2					U				
8.9								GA	
8.1									T
7.7	AG								
7.5					U				
7.4	AG								
7.3				TG					
7.2		A							
6.3					G				
5.7									T
5.6								G	
5.5									T
5.3						G			
5.1			AG						
4.6						G			
4.4		A							

4.0	A								
3.8		A							
3.4			A						
3.4								G	
3.3							GA		
2.7						G			
2.5	A								
2.2							GA		
1.8		A							
1.8				U					
1.7		A							
1.5								G	
1.4			A						
1.4	A								
1.3							GA		
1.2			A						
1.1	A								
1.0							GA		
0.9		A							

0.7				TG					
0.5							GA		
0.4							G		
0.3							G		
0.0	A	A	A	T	G	G	G	G	T

Nucleotide base calls for specific mtDNA mutations by MALDI-TOF MS (Sequenom™, San Diego, CA). Test samples were generated from cloned mtDNA from one subject, and mixed in varying proportions. For nine mutations, the percentage mutated mtDNA in the mixed clone sample was determined by last-cycle fluorescent PCR (Materials and Methods). The detection threshold for the m.1555A>G could not be quantified due to lack of an efficient fluorescent RFLP assay. However, dilution curves similar to those demonstrated estimated the assay to be efficient in detecting low levels of heteroplasmy. Quantification by cloning and sequencing mtDNA fragments of the lowest dilution positively identified by MALDI-TOF MS indicated the threshold of detection for m.1555A>G was 1.4% (1 of 70 independent clones). Coloured shading (pink, yellow, orange and blue) indicates the mutations grouped together in a multiplex primer extension reaction. Green shading = heteroplasmy levels where the mutated base was detected by MALDI-TOF MS, with the base call as specified.

Table S2. Oligonucleotide Sequences Used for Quantification of Mitochondrial Heteroplasmy

Mutation	Primer Position (np)	Restriction site (np / enzyme)	RFLP positive	RFLP negative
m.3243A>G	3155-3171<br>3353-3334	+3244 *Hae III*	90, 72, 37	162, 37
m.8993T>G	8961-8980<br>9100-9081	+ 8991 *Hpa II*	109, 31	140
m.13513G>A	13491-13512<br>13610-13592	-13522 *MboII*	120	88, 32
m.1555A>G	1266-1282<br>1588-1569	-1546 *BsmAI*	323	281, 42
m.8344A>G	8191-8212<br>8390-8345	+8350 *BglI*	160, 40	200
m.3460G>A	3397-3419<br>3560-3538	-3458 *BsaHI*	147, 17	102, 17, 45
m.11778G>A	11618-11637<br>12009-11990	+11774 *Tsp45I*	157, 188, 47	345, 47
m.14459G>A	14265-14282<br>14614-14590	-14439 *BtgZI*	250, 101	175, 101, 75
m.7445A>G	7408-7427<br>7548-7571	- 7441 *Hpy188III*	164	130, 34
m.14484T>C	14460-14483<br>14675-14694	-14481 *MboI*	235	213, 22

Np = nucleotide positions refer to the revised Cambridge reference sequence (rCRS)[1]. + = restriction site gain. - = restriction site loss. Fragment lengths detectable by fragment analysis on the Beckman Coulter CEQ™ 8000 are displayed as base pairs in length, highlighted in green.

Table S3. Oligonucleotide Sequences for Sequencing of mtDNA D-Loop

Name	Sequence	Primer Position (np)
D1	F5’ TGTAAAACGACGGCCAGTATCGGAGGACAACCAGTAAG3’	15758- 15777
	R5’ CAGGAAACAGCTATGACCTGGGTAGGTTTGTTGGTATC3’	16294- 16274
D2	F5’TGTAAAACGACGGCCAGTCTCAACTATCACACATCAACTG3’	16223- 16244
	R5’ CAGGAAACAGCTATGACCAGATACTGCGACATAGGGTG3’	129- 110
D3	F5’TGTAAAACGACGGCCAGTTATTAACCACTCACGGGAGC3’	20-39
	R5’ CAGGAAACAGCTATGACC CTGGTTAGGCTGGTGTTAGG3’	389- 370
D4	F5’TGTAAAACGACGGCCAGTGCCACAGCACTTAAACACATC3’	323- 343
	R5’ CAGGAAACAGCTATGACCTGCTGCGTGCTTGATGCTTG3’	771- 752

Np = nucleotide positions refer to the revised Cambridge reference sequence (rCRS).[1]

Supplemental Reference

1. Andrews RM, Kubacka I, Chinnery PF, Lightowlers RN, Turnbull DM, Howell N. Reanalysis and revision of the Cambridge reference sequence for human mitochondrial DNA [letter]. Nat Genet. 1999;23:147.
